# Development of an Ex Vivo Osteochondral Biomimetic Platform for Mechanistic Investigation of Cartilage Regeneration

**DOI:** 10.3390/ijms262311759

**Published:** 2025-12-04

**Authors:** Johanna Brobeil, Dorothea Alexander, Felix Umrath, Marina Danalache

**Affiliations:** 1 Laboratory for Cell Biology, Department of Orthopedic Surgery, University Hospital of Tübingen, Waldhörnlestr. 22, 72072 Tübingen, Germany; johanna.brobeil@student.uni-tuebingen.de (J.B.);; 2 Department of Oral Maxillofacial Surgery, University Hospital of Tübingen, 72072 Tübingen, Germany; dorothea.alexander@med.uni-tuebingen.de

**Keywords:** osteochondral model, cartilage regeneration, collagen deposition, MSC, dual-compartment culture, matrix remodeling dynamics

## Abstract

Articular cartilage possesses limited intrinsic healing capacity due to its avascular and aneural nature, posing a significant challenge for treating focal chondral defects. While regenerative strategies employing biomaterials and stem cells have progressed, their mechanistic evaluation is hindered by the lack of physiologically relevant in vitro models. This study aimed to establish and characterize a human ex vivo osteochondral explant model to assess cellular and extracellular matrix responses to cartilage repair strategies. Osteochondral explants (10 mm diameter, *n* = 61) were harvested from femoral condyles of patients undergoing knee arthroplasty. Standardized full-thickness chondral defects (4 mm) were created and assigned to six treatment groups: native control, untreated defect, fibrin glue, collagen type I hydrogel (ChondroFiller^®^), fibrin glue + MSCs, and ChondroFiller^®^ + MSCs. Explants were cultured for 7, 14, or 21 days, followed by metabolic, biochemical, and histological assessments. Explants remained viable for 21 days. Notably, the ChondroFiller^®^ group showed a 2.4-fold increase in DNA content by day 14, while MSC-treated groups enhanced collagen deposition and GAG production. Significant correlations between DNA and collagen levels were observed in scaffold-based treatments. This ex vivo model offers a reproducible and translational platform to investigate cartilage regeneration with temporal resolution, supporting preclinical testing of emerging therapeutic approaches.

## 1. Introduction

Articular cartilage plays a vital role in load transmission and frictionless joint movement, yet its intrinsic repair capacity is severely limited due to its avascular, aneural, and alymphatic nature [1]. Consequently, focal chondral defects, whether traumatic or degenerative, present a significant clinical challenge and are frequently implicated in the progression of joint pathology [2]. Traditional surgical interventions for focal chondral defects-including microfracture, osteochondral autograft transplantation, and autologous chondrocyte implantation, have demonstrated variable clinical success [3] and are frequently limited by suboptimal tissue integration and regeneration quality [4].

In recent years, regenerative medicine strategies have gained prominence, particularly those aiming to restore native tissue structure by stimulating the body’s endogenous repair mechanisms. Within this broader field, tissue engineering represents a distinct and more targeted approach. Formally classified as an Advanced Therapy Medicinal Product (ATMP) under regulatory frameworks, tissue engineering typically is a combination of cells (e.g., mesenchymal stem cells (MSCs)), biomaterials, and sometimes signaling molecules to construct implantable tissue constructs that facilitate regeneration upon transplantation [5]. Among these, biomaterials such as collagen-based or hydrogel scaffolds offer a supportive matrix that facilitates cellular attachment, proliferation, and differentiation, particularly when used in conjunction with MSCs capable of chondrogenic differentiation [6,7]. However, despite promising in vitro outcomes, many of these strategies fail to achieve consistent translational success due to the lack of standardized, mechanistically informative in vitro models [8].

Animal models, while indispensable for understanding whole-organism responses, often fall short in replicating human-specific cellular and matrix interactions, particularly at the osteochondral interface. Small animal models, such as mice and rabbits, have thin cartilage layers and small joint sizes, which limit their translational relevance due to differences in cartilage thickness and intrinsic healing capacity compared to humans [9]. Larger animal models like goats, pigs, and horses provide thicker cartilage and more anatomically comparable joints but still differ in subchondral bone remodeling, joint loading, and matrix composition, which impacts repair outcomes [9,10]. Conventional in vitro chondrocyte monolayer or spheroid cultures are similarly limited, lacking the native zonal architecture and mechanical environment of the joint [11].

Human ex vivo osteochondral explants (OEs), which preserve native tissue architecture and biochemical gradients, offer a compelling solution [12], emerging as promising platforms for evaluating regenerative therapies under controlled and physiologically relevant conditions [13,14]. To this end, we developed and tested an ex vivo human-based OE model to evaluate the biological efficacy of biomaterial-based and MSC-assisted cartilage repair strategies. By introducing reproducible full-thickness chondral defects and stratifying explants across multiple treatment arms and timepoints, our model facilitates comprehensive assessment of cellular viability, matrix composition, and histological outcomes. In particular, we characterize the utility of this model for testing six therapeutic strategies: untreated defects, fibrin glue, ChondroFiller^®^ (meidrix Biomedicals GmbH, Esslingen, Germany, which is a clinically available collagen type I-based scaffold), and their combinations with MSCs. Through longitudinal analysis of metabolic activity, extracellular matrix (ECM) synthesis, and cellular dynamics, we aimed to pinpoint the validity of our model to assess treatment-specific repair mechanisms.

## 2. Results

### 2.1. Evaluation of Cellular Viability in OEs Using MTT Staining

**Figure 1:**
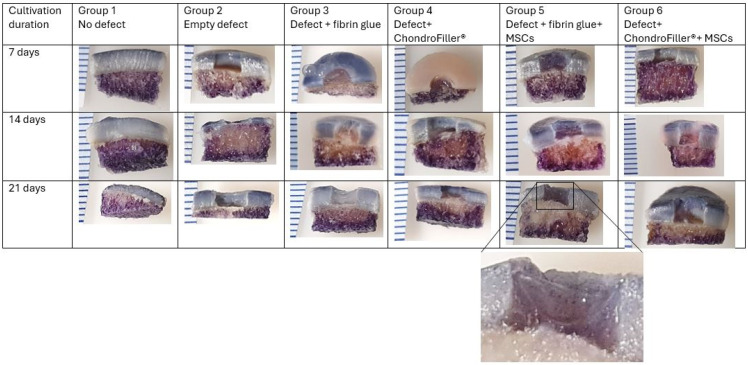
Overview of MTT-stained osteochondral explants (OEs) of all groups and all time points. OEs were cultured in a dual chondral-subchondral compartment system and analyzed at defined time points: day 7 (first row), day 14 (second row), and day 21 (third row). Magnified views of the defect area reveal viable MSCs, indicated by dark purple staining, persisting up to day 21. The intensity of MTT staining reflects mitochondrial activity, thereby confirming sustained metabolic activity and cell viability within the construct over the culture period. Distance between two lines (left side): 2 mm.

Cell viability was evaluated using the MTT assay at three defined time points: d7, d14, and d21 of cultivation (Figure 1).

At all timepoints, MTT staining yielded positive results, confirming the presence of viable, metabolically active cells. A notably higher staining intensity was observed at d7 in the subchondral bone region compared to the overlying cartilage, suggesting elevated mitochondrial activity or cell density in the bone compartment (first row). However, a progressive decline in the overall MTT staining intensity was evident in the subchondral region over the cultivation period (i.e., d21), particularly in the central zone, which may reflect either a reduction in metabolic activity or cell viability over time (second and third row). Concurrently, the viability of the MSCs which were filled into the defect was assessed at all designated time points (d7, d14, and d21, respectively). Despite the prolonged in vitro conditions, cells remained metabolically active up to d21, as evidenced by sustained MTT staining. This indicates that the MSCs retained viability throughout the experimental period (magnified view, subgroup 5, third row).

### 2.2. Histological Evaluation Confirms MSC Retention, Scaffold Integration, and Early Matrix Deposition

**Figure 2:**
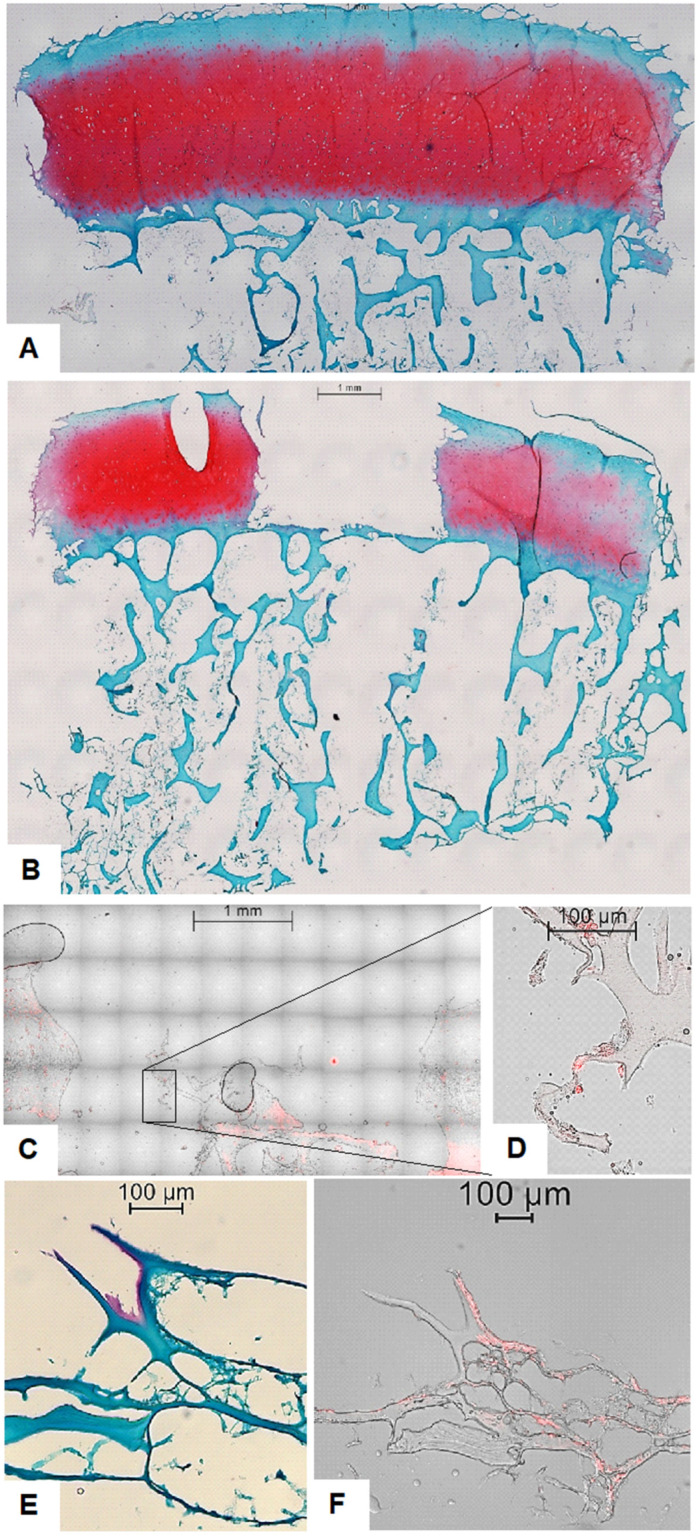
Overview of histological and fluorescence microscopy images of osteochondral explants (OEs). (**A**) Safranin-O/Fast Green/Hematoxylin staining depicting the overall matrix structure and cellular morphology within an OE without a defect. Scale bar 1 mm. (**B**) Safranin-O/Fast Green/Hematoxylin staining depicting the overall matrix structure and cellular morphology within an OE with an empty defect. Scale bar 1 mm. (**C**) Fluorescence overlay of brightfield and propidium iodide (PI) staining of osteochondral explant (OE) containing a ChondroFiller^®^-filled defect without exogenously added MSCs at 20× magnification, illustrating red-fluorescent nuclei of endogenously migrated cells within the scaffold. Scale bar 1 mm. (**D**) High-resolution overlay image (40× magnification) confirming the presence and nuclear localization of cell nuclei, in the absence of additional cell (i.e., MSC) supplementation. Scale bar 100 µm. (**E**) Histological analysis of OEs with defect filled using ChondroFiller^®^ and MSC. Staining at 40× magnification. The ChondroFiller^®^ formed a densely branched network within the defect site, providing a provisional matrix for cellular infiltration. In some regions, Safranin-O positive areas were detected near MSCs, suggesting localized GAGs deposition and early matrix synthesis. Scale bar 100 µm. (**F**) Fluorescence overlay of brightfield and propidium iodide (PI) staining of osteochondral explants (OEs) with defect filled using ChondroFiller^®^ and MSC at 40× magnification, illustrating red-fluorescent nuclei of supplemented MSCs. Cells were still visibly embedded within the collagen matrix after 21-day cultivation. Scale bar 100 µm.

Histological analysis was employed to visualize the spatial organization of implanted cells and the structural characteristics of the defect filling materials. Results confirmed the presence and localization of cells within the defect. Fluorescence microscopic analysis identified the presence of endogenously migrated cells within OE defect regions in subgroups lacking exogenously applied MSCs (Figure 2C,D). This was demonstrated by propidium iodide (PI) staining, wherein cell nuclei exhibited intense red fluorescence under excitation, confirming nuclear localization (Figure 2D).

On the other hand, in OEs treated with fibrin glue and MSCs, histological sections stained with hematoxylin, Safranin-O, and Fast Green revealed a dense, highly branched network occupying the defect region. Cells were clearly embedded within this network, indicating successful cellular incorporation into the scaffold matrix. Notably, several OEs exhibited localized red staining with Safranin-O, a hallmark of GAG deposition, in proximity to the implanted MSCs. This observation suggests the initiation of early de novo extracellular matrix formation (Figure 2E,F).

### 2.3. Temporal Changes in Cellularity and DNA Content (Hoechst Assay) over the Culture Period

**Figure 3:**
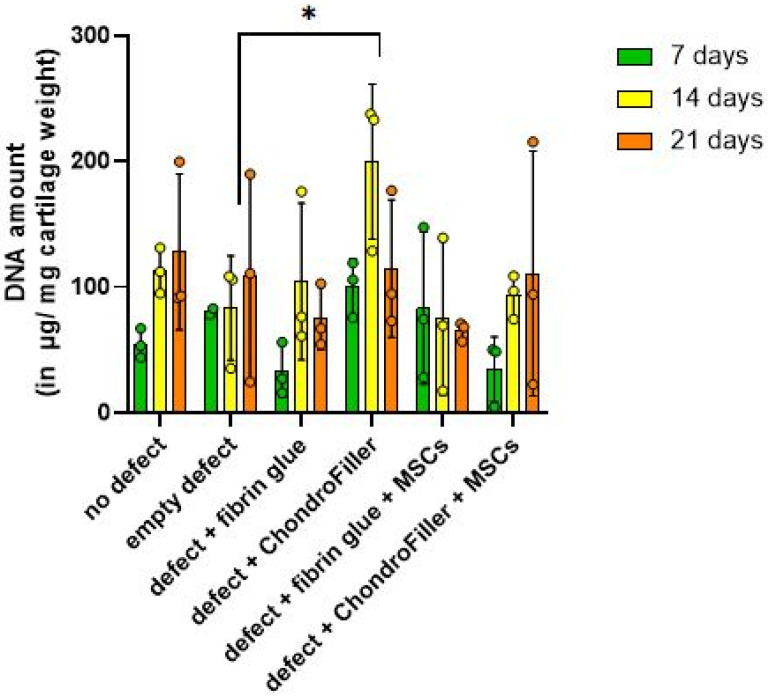
Quantitative assessment of DNA content in osteochondral explants (OEs) across six experimental groups over a 21-day in vitro cultivation period. DNA was measured at d7, 14, and 21 to evaluate cellularity and potential proliferative responses. The experimental groups included: i ntact OEs without defects, OEs with untreated defects, defects filled with fibrin glue, defects treated with a type I collagen-based hydrogel (ChondroFiller^®^), defects filled with fibrin glue and human bone marrow-derived MSCs, defects treated with ChondroFiller^®^ combined with MSCs. Data (*n* = 3 per group) are presented as *mean ± SD*. Statistical analysis was performed using two-way ANOVA, * = *p <* 0.05.

DNA content within the cartilage regions of the OEs was evaluated at a culture timepoints of d7, 14, and 21 (Figure 3). In OEs with defects filled with ChondroFiller^®^, the DNA amount increased compared with the empty-defect group at d14. Thereafter, the values declined again and reached lower levels at d21.

DNA quantification over the cultivation period revealed a biphasic pattern in cellular content within OEs. A modest reduction in total DNA content was detected at day 7, potentially reflecting an initial cellular stress response, limited proliferation, or partial cell loss post-seeding. This was followed by a marked increase in DNA content by d14, suggestive of enhanced cell proliferation or improved cellular retention within the scaffold microenvironment. Notably, in subgroup 4, comprising defects treated with ChondroFiller^®^, the increase in DNA content at day 14 was statistically significant (*p* = 0.0108, 95% CI = [−253.0, −26.74]), corresponding to an approximately threefold elevation relative to the untreated (empty defect) control group. The control group served as a baseline to which the treatment groups were compared to. By d21 time point, DNA content exhibited a decline across experimental groups except the group 6 (ChondroFiller^®^ + MSC). These observations highlight a temporal dynamics pattern of cells within the engineered osteochondral environment.

### 2.4. Transient Restoration of GAG Content in OEs Filled with ChondroFiller^®^

**Figure 4:**
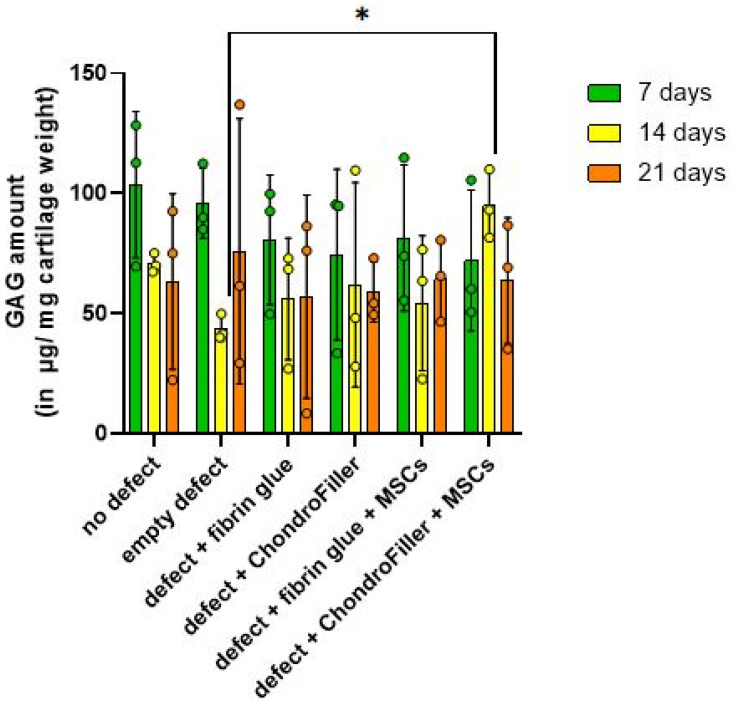
Quantitative assessment of GAG content in osteochondral explants (OEs) across six experimental groups over a 21-day in vitro cultivation period. GAG was measured at d7, 14, and 21 via DMMB assay. The experimental groups included: intact OEs without defects, OEs with untreated defects, defects filled with fibrin glue, defects treated with a type I collagen-based hydrogel (ChondroFiller^®^), defects filled with fibrin glue and human bone marrow-derived MSCs, defects treated with ChondroFiller^®^ combined with MSCs. Data (*n = 3* per group) are presented as *mean ± SD*. Statistical analysis was performed using two-way ANOVA, * = *p* < 0.05.

GAG content within the cartilage regions of the OEs was evaluated at d7, 14, and 21 using the DMMB assay (Figure 4).

A reduction in GAG concentration was observed across all groups by d7, suggesting an early depletion of ECM components following in vitro cultivation. Notably, by d14, OEs comprising defects treated with ChondroFiller^®^ in combination with MSCs, exhibited a statistically significant increase in GAG content compared to the untreated defect group (*p* = 0.0130, 95% CI = [−212.8, −20.10]). At d21, GAG levels declined again, possibly due to matrix degradation or reduced biosynthetic activity under extended culture conditions. These findings suggest that the combination of ChondroFiller^®^ and MSCs temporarily supports ECM production, particularly during the intermediate phase of culture.

### 2.5. Hydroxyproline Levels Reveal a Temporary Surge in Matrix Accumulation with ChondroFiller^®^

**Figure 5:**
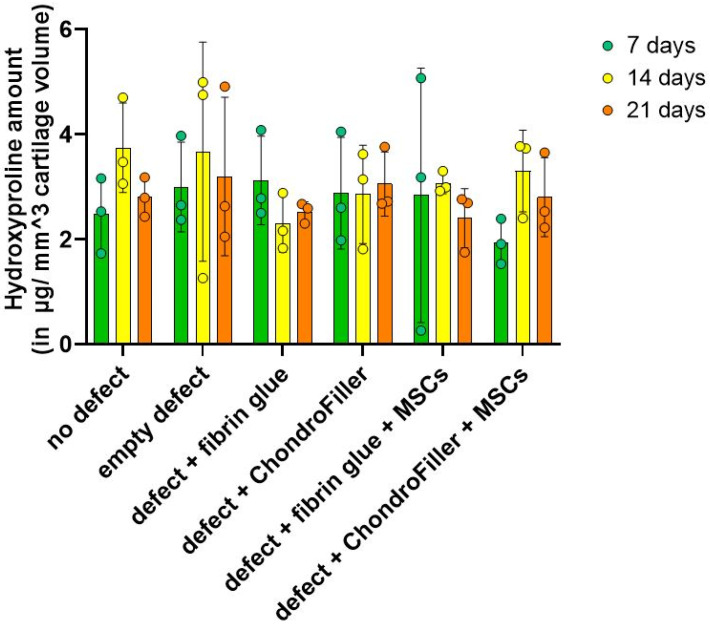
Quantification of hydroxyproline content in osteochondral explants (OEs) as an indirect measure of total collagen deposition in OEs constructs over a 21-day culture period. Hydroxyproline levels were assessed at days 7, 14, and 21 across six experimental groups: intact OEs without defects, OEs with untreated defects, defects filled with fibrin glue, defects treated with a type I collagen-based hydrogel (ChondroFiller^®^), defects filled with fibrin glue and human bone marrow-derived MSCs, defects treated with ChondroFiller^®^ combined with MSCs. Data (*n* = 3 per group) are presented as *mean ± SD*.

Hydroxyproline content, used as a biochemical surrogate for total collagen accumulation, was quantified over the d21 in vitro cultivation period. A modest increase in hydroxyproline levels was observed by d14, indicating enhanced collagen synthesis within the engineered OEs (Figure 5).

It should be noted that the collagen (hydroxyproline) content observed in the ChondroFiller^®^ groups may not necessarily indicate exclusively increased endogenous matrix synthesis but could instead be attributed to the exogenously supplied type I collagen inherently present in the scaffold material. However, across all groups, by d21 the hydroxyproline content declined.

### 2.6. Biochemical Interdependence Between Cellularity and Collagen Deposition

**Figure 6:**
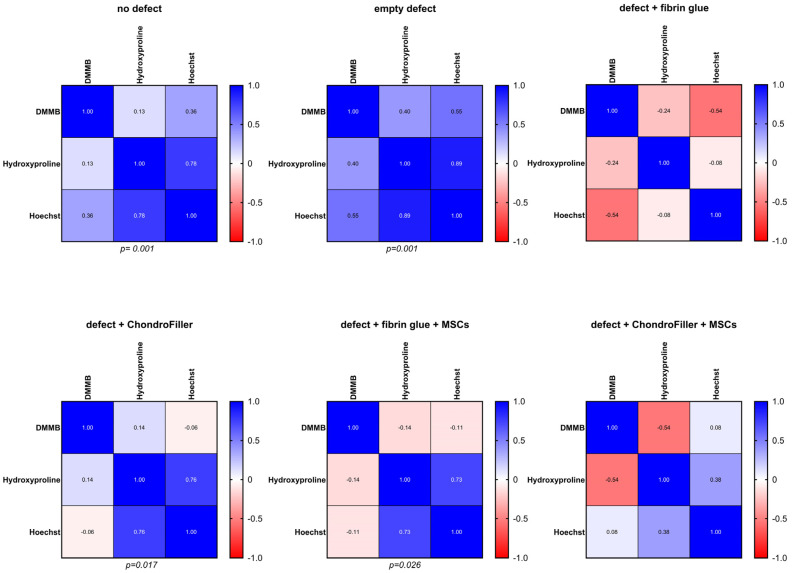
Correlation analysis between DNA content and hydroxyproline levels across osteochondral explants (OEs) for all experimental groups. The six experimental groups were: intact OEs without defects, OEs with untreated defects, defects filled with fibrin glue, defects treated with a type I collagen-based hydrogel (ChondroFiller^®^), defects filled with fibrin glue and MSCs, defects treated with ChondroFiller^®^ combined with MSCs. Statistical significance and correlation coefficients were calculated using Pearson correlation. Significance level defined at *p* < 0.05.

To evaluate potential biochemical interdependence, correlation analyses were performed between DNA content (cellularity) and hydroxyproline levels (collagen content) across the experimental subgroups. The results are visualized as correlation matrices, where the color intensity reflects the strength and direction of correlation coefficients: deep blue denotes strong positive correlations (r approaching +1), red indicates negative correlations (r approaching −1), and lighter shades represent weaker associations (Figure 6). In the no defect and empty defect groups, strong positive correlations were observed between DNA and hydroxyproline content (r = 0.78 and r = 0.89, respectively; *p* < 0.001), along with moderate positive correlations between DNA and glycosaminoglycan (GAG) content (r = 0.36 and r = 0.55, respectively). These results suggest that cellularity is positively associated with ECM integrity, where increased cell presence correlates with enhanced collagen and GAG content, while reduced cellularity is linked to compromised matrix composition. In contrast, the defect + fibrin glue and defect + ChondroFiller^®^ groups exhibited more variable and, in some cases, inverse correlation patterns. For instance, a moderate negative correlation was observed between DNA and GAG in the fibrin glue group (r = −0.54), indicating that higher cell density did not necessarily correspond to improved matrix deposition in these conditions. In the ChondroFiller^®^ + MSCs group, only mild positive correlations were found between DNA and hydroxyproline (r = 0.28, light blue), whereas a negative correlation was observed between DNA and GAG content (r = −0.54, red hues). These results suggest that osteochondral regeneration depends on a tightly regulated interplay between cellular density, activity and matrix synthesis. The treatment-dependent correlation patterns emphasize the importance of scaffold–cell interactions in guiding effective tissue repair.

## 3. Discussion

This study aimed to investigate the utility and translational relevance of a human ex vivo OE model for dissecting the temporal and spatial dynamics of cartilage repair strategies. By employing a dual-compartment culture system and standardized defect induction, we were able to systematically evaluate the effects of biomaterial scaffolds and MSC supplementation on cellular viability, proliferation, and matrix remodeling within a structurally intact osteochondral unit. Our results demonstrated robust bulk viability at all examined time points, indicating sustained metabolic activity within the constructs. Notably, staining intensity was highest at d7 in the subchondral bone region relative to the overlying cartilage. However, a gradual reduction in the overall bulk staining was observed by d21, particularly in the central zone of the subchondral compartment. This decline could reflect decreased cellular activity due to nutrient depletion, hypoxia, or cell senescence under static culture conditions, as previously described in OEs models [15,16]. Notably, the added MSCs remained metabolically active throughout the 21-day culture period, as confirmed by sustained MTT staining as well as histological staining. These findings underscore the resilience and adaptability of human MSCs within 3D scaffold environments, even in the absence of exogenous growth factors or mechanical stimulation. Previous studies have reported similar persistence of MSC viability in hydrogel-based constructs, highlighting their potential for long-term regenerative applications [16,17,18]. MSCs are well recognized for their secretion of a diverse repertoire of trophic factors including cytokines, growth factors, and extracellular vesicles, that modulate host cell recruitment, stimulate ECM production, and exert potent immunomodulatory effects [6,19]. This aspect is further substantiated by our histological analyses, which revealed that MSCs not only maintained metabolic activity throughout the 21-day culture period, as evidenced by persistent MTT staining, but were also spatially associated with focal de novo GAG accumulation within the defect regions. Especially in OEs with ChondroFiller^®^ + MSC groups, cellular localization within the defect area, with red fluorescent nuclei visible via cell nuclear staining together with hematoxylin/Safranin-O/Fast Green staining demonstrated the formation of a branched collagen networks embedding the MSCs, and localized red Safranin-O staining around the cells indicated early GAG production. This co-localization suggests that the implanted MSCs retained functional relevance during the early phases of matrix remodeling, actively contributing to nascent ECM synthesis. Our qualitative data are in line with reports showing de novo matrix synthesis suggesting early stages of chondrogenic differentiation [20,21]. However, it is important to acknowledge that the cellular identity of the matrix-producing cells could not be unequivocally determined within the scope of this model. While the spatial and temporal association of implanted MSCs with zones of de novo matrix deposition is suggestive, we cannot rule out the contribution of endogenously recruited or migratory chondrocytes [22] that may have infiltrated the defect site. Consequently, to delineate the specific cellular contributions to matrix formation, future investigations should incorporate lineage tracing methodologies [23], fluorescent or genetic cell labeling, and molecular profiling techniques. Additionally, it should be taken into account that while MTT staining provided a qualitative approximation of metabolic activity, its application in thick 3D constructs is constrained by limited reagent diffusion, heterogeneous formazan accumulation, and optical scattering [24,25]. Therefore, future studies should prioritize soluble viability assays, such as ATP quantification or secreted reporter enzyme analyses, which overcome diffusion barriers and allow for more reliable and representative assessment in high-density tissue models [26].

DNA quantification demonstrated a biphasic response, with an initial decrease in cellularity at d7, followed by recovery at d14. This pattern is consistent with the post-seeding adaptation phase commonly observed in 3D cultures [27]. Notably, the ChondroFiller^®^ group showed a significant increase in DNA content by d14, suggesting that collagen-based scaffolds can enhance cell retention and proliferation. This observation is supported by previous findings highlighting the pro-survival and integrin-mediated adhesion properties of type I collagen hydrogels [28,29]. However, by d21, a decline in DNA content was observed across all groups, potentially reflecting nutrient depletion, contact inhibition, or onset of senescence in static culture conditions. Similarly, GAGs quantification revealed a significant increase at d14 in the type I collagen scaffold (ChondroFiller^®^) + MSC group reaching nearly native tissue levels (94.91 vs. 110.03 µg/mg cartilage). This observation aligns with findings from studies that reviewed the use of MSCs embedded in biomimetic hydrogels such as type I collagen-based scaffolds, which reported enhanced proteoglycan synthesis [30,31]. Similar positive outcomes have been reported in an in vivo rabbit model, where type I and type II collagen-based hydrogels were evaluated for the treatment of focal cartilage defects, demonstrating effective integration and enhanced matrix regeneration within the defect site [32]. The chondrogenic medium itself may contribute to the observed GAG release by MSCs residing in the collagen scaffold. Although ChondroFiller^®^ alone modestly increased hydroxyproline content (a collagen proxy), it lacked the robust GAG production observed with cellular supplementation, reaffirming that scaffold chemistry alone is insufficient to induce complete matrix regeneration.

Correlation analysis revealed how different defect repair strategies influence the coordination between cellularity and matrix formation. In the empty defect group, quantitative analyses were performed on the residual cartilage tissue, which consisted exclusively of native chondrocytes. Given that biochemical parameters were normalized to the wet weight of the cartilage (DNA and GAG) or the tissue volume (hydroxyproline), the observed strong positive correlations between DNA content, GAG concentration, and hydroxyproline levels provide direct evidence that higher cell density is associated with increased extracellular matrix (ECM) synthesis [33,34]. This relationship is consistent with the anabolic function of chondrocytes in maintaining the ECM in healthy cartilage [35]. Conceptually, the biochemical profile of the empty defect group should approximate that of the intact, non-defected osteochondral explant, as the intervention involved only the mechanical removal of a defined volume of cartilage, containing both cells and ECM without altering the intrinsic cell concentration. This tissue removal was proportionally accounted for in both mass normalization and subsequent biochemical quantification. ChondroFiller^®^-filled OE defects also showed strong correlations, indicating that collagen-based scaffolds support both cell viability and matrix production in a coordinated manner. This suggests that infiltrating cells presumably chondrocytes or progenitor cells undergoing chondrogenic differentiation actively contribute to matrix production within the scaffold. Similar findings have shown collagen hydrogels to provide a biomimetic environment that facilitates cell–matrix interactions, enhancing chondrogenic responses [36]. Moreover, clinical research showed that collagen type I biomaterials integrate well with native tissue, showing good graft attachment and cartilage regeneration with smooth surfaces and homogenous repaired tissue structure over time [37]. However, it must be considered that the ChondroFiller^®^ itself contains substantial quantities of exogenous type I collagen, thereby contributing directly to the hydroxyproline signal. As a result, distinguishing scaffold-derived collagen from newly synthesized ECM is challenging, and this background signal must be considered when interpreting absolute collagen levels and the extent of de novo matrix deposition. Future studies should incorporate methods such as stable isotope labeling and proteomic analysis to accurately distinguish scaffold-derived collagen from host-synthesized ECM components [38,39]. Nevertheless, under such conditions, one might have expected a diminished correlation, as the scaffold introduces hydroxyproline independently of cellular activity. The persistence of a strong correlation despite this exogenous input suggests that the cellular component of the repair tissue was sufficiently robust to offset the potential dilution effect, likely due to effective cell migration and integration into the scaffold, accompanied by active matrix synthesis. Interestingly, OEs defects filled with fibrin glue + MSC demonstrated a more variable correlation profile, including both weak and negative associations, particularly between DNA and matrix content. This may indicate biological heterogeneity in cellular response, and cell–scaffold dynamics. MSCs exhibit high plasticity and their function is modulated by matrix stiffness, and composition [40,41], while fibrin fixation can hamper cellular infiltration and proliferation of MSC [42]. It has to be noted that the correlations in all groups remain weaker than those observed in the no defect group, indicating incomplete restoration of tissue homeostasis during the culture time of 21 days.

Overall, our model employed patient-derived human osteochondral explants from total knee arthroplasty patients, incorporating standardized full-thickness defects and six distinct treatment arms, enabling controlled, head-to-head comparisons of regenerative strategies. It should be noted that although no visible cross-compartment leakage was observed in our dual-compartment system, quantitative validation of barrier integrity was not performed. While removal of the insert membrane was intended to replicate physiological bone–cartilage continuity, this approach may reduce strict compartmental separation. Future studies should incorporate objective measures, such as FITC-dextran flux assays, to assess barrier function and paracellular transport dynamics.

While Abbas et al. previously emphasized the synergistic effects of MSCs and cartilage fragments in similar OEs, their model was limited to two treatment groups and lacked a spatially controlled environment that differentiates between bone and cartilage compartments [43]. Similarly to the dual-compartment setup on bovine OEs introduced by de Vries-van Melle et al. [44], our model preserves the native cartilage-bone interface and enables compartment-specific culture conditions. It is important to note that all ex vivo models, including ours share certain limitations such as limited duration of culture (with our model validated up to 21 days), the lack of systemic influences such as immune and vascular responses, and the inherent variability of human donor tissue. While cartilage matrix composition has been shown to remain stable for more than four weeks in ex vivo culture, as demonstrated by Schwab et al. [12], consistent and reproducible outcomes depend heavily on careful explant selection and monitoring. Although some advanced models integrate mechanical stimulation or inflammatory challenges to better mimic in vivo conditions [45,46,47], these approaches introduce substantial complexity and are not yet standardized in preclinical cartilage repair studies. To robustly analyze the regenerative dynamics and account for biological variability, future studies should incorporate increased sample sizes and extended culture durations, thereby enhancing statistical power and enabling more definitive conclusions regarding scaffold efficacy and tissue remodeling outcomes. Furthermore, future studies should also seek to mitigate donor heterogeneity by prospectively balancing key biological covariates, particularly sex and age and clinically relevant characteristics [48], across experimental groups and by standardizing tissue procurement and culture protocols to reduce avoidable technical variability.

## 4. Materials and Methods

### 4.1. Preparation of Osteochondral Explants (OEs) from Human Donors

Femoral condyle tissue was collected from 17 patients (6 males, 11 females; mean age 64.4 years) undergoing total knee arthroplasty for primary osteoarthritis. Institutional and ethical approval and patients’ consent were obtained before the commencement of the study (project number 672/2016BO2). Individuals with a history of trauma, immunological, or neoplastic disorders were excluded. Resected joints were immediately transferred to high-glucose Dulbecco’s modified Eagle’s medium (DMEM; Gibco, Life Technologies, Darmstadt, Germany) supplemented with penicillin (100 U/mL), streptomycin (100 µg/mL), and amphotericin B (1%, *v*/*v*). Using a custom-fabricated coring device, 61 cylindrical OEs (Ø 10 mm; bone depth ≤ 5 mm) were isolated from regions with macroscopically intact cartilage and rinsed in sterile PBS. From each patient, a total of three to nine OEs were harvested from femoral regions.

### 4.2. Chondral Defect Induction and Experimental Design

Standardized full-thickness chondral defects (4 mm diameter, extending to the tidemark) were manually induced at the center of each 10 mm OE using a sterile biopsy punch. Defect sites were carefully cleaned of residual debris using sterile tweezers and a scalpel under aseptic conditions.

**Figure 7:**
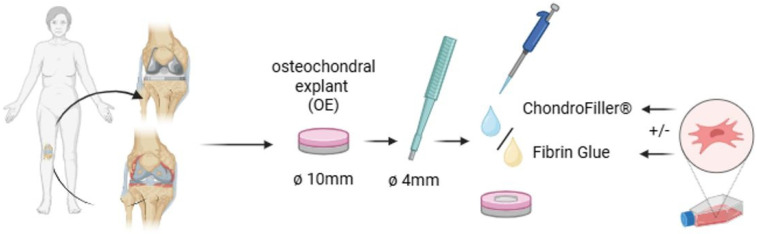
Experimental workflow for the preparation and treatment of osteochondral explants (OEs). OEs (Ø 10 mm) were harvested from the femoral condyles of human knee joints. Standardized full-thickness chondral defects (Ø 4 mm) were introduced centrally using a biopsy punch. Defects were left untreated or filled with either fibrin glue or a collagen type I -based hydrogel (ChondroFiller^®^), with or without the addition of human bone marrow-derived mesenchymal stem cells (MSCs; 5 × 10^4^ cells/defect). Explants were then cultured for predetermined time points (7, 14, and 21 days, respectively) under defined conditions for subjected to subsequent analysis (created with BioRender, www.biorender.com).

The explants were stratified into six experimental groups (*n* = 9 per group): (1) intact OEs without defects, (2) untreated defects, (3) defects filled with fibrin glue, (4) defects filled with a collagen type I-based gel (ChondroFiller^®^, meidrix Biomedicals GmbH, Esslingen, Germany), (5) defects filled with fibrin glue and 5 × 10^4^ human bone marrow-derived mesenchymal stem cells (MSCs, ethical approval number for MSCs: 885/2021BO2), and (6) defects filled with ChondroFiller^®^ combined with 5 × 10^4^ MSCs. Each group was subdivided equally across three culture time points: 7, 14, and 21 days (d7, d14, d21; *n* = 3 per time point per condition). For the cell-based treatment conditions, defects were filled with 25 µL of a fibrin or ChondroFiller^®^ matrix pre-mixed with 5 × 10^4^ MSCs per explant. A representative flow chart of the experimental approach is shown in Figure 7.

To mimic the physiological distinction between cartilage and subchondral bone environments, OEs were cultured in a two-compartment system. Explants were secured in 12-well ThinCert^®^ cell culture inserts (Greiner Bio-One, Kremsmünster, Austria) and stabilized using fibrin glue to ensure both mechanical fixation and compartmental sealing. Following complete polymerization of the fibrin, the insert membrane was carefully removed to allow direct communication with the basal compartment. The fibrin glue functioned as a physical barrier between the cartilage and bone regions. This configuration enabled differential media exposure while maintaining physiological, native continuity between tissue regions. The inserts were then transferred into deepwell 12-well plates (Greiner Bio-One), creating a vertically oriented dual-culture interface. The upper (chondral) compartment was filled with a cartilage-specific medium comprising high-glucose DMEM (Gibco) supplemented with 100 U/mL penicillin, 100 µg/mL streptomycin, 1% (*v*/*v*) amphotericin B, 1% (*v*/*v*) ITS+ Premix (BD Biosciences, Franklin Lakes, NJ, USA), 100 nM dexamethasone, 1 µM L-ascorbic acid-2-phosphate, and 10 ng/mL recombinant human TGF-β1 (all from Sigma-Aldrich, St. Louis, MO, USA) as previously described by Salchaga et al. [49]. The lower (osseous) compartment was supplied with MSC medium consisting of high-glucose DMEM/F-12 (Gibco) supplemented with 10% (*v*/*v*) human platelet lysate, 100 U/mL penicillin, 100 µg/mL streptomycin, and 1% (*v*/*v*) amphotericin B (Sigma). Separation between the two compartment was assessed during each media change (twice a week) by visual inspection, and no cross-compartment leakage was observed throughout the culture period.

Each experimental subgroup was evenly distributed across three culture durations: 7 (d7), 14 (d14), and 21 (d21) days with three OEs allocated to each time point. Upon completion of the culture period, OEs (*n* = 61) were longitudinally bisected in halves under sterile conditions. The cartilage compartment, including any applied defect treatment, was carefully dissected from the subchondral bone for targeted downstream analyses.

### 4.3. Viability Assessment

To evaluate cell viability in the OEs after culturing, 3-(4,5-dimethylthiazolyl-2)-2,5-diphenyltetrazolium bromide (MTT staining; Sigma) was used. A 5 mg/mL solution of MTT in PBS was diluted to a concentration of 0.37 mg/mL with DMEM (Gibco), and OE halves were incubated for 90 min at 37 °C. Macroscopic pictures were taken after washing with PBS. Fresh OEs served as positive controls.

### 4.4. Safranin-O/Fast Green/Hematoxylin/Propidium Iodide

Half of the 43 OEs were initially fixed in 4% (*v*/*v*) formalin in PBS for one to three days, followed by decalcification in 10% (*v*/*v*) formic acid for seven days. After a second fixation in 4% (*v*/*v*) formalin, they were embedded in Tissue-Tek (Sakura, Osaka, Japan) and frozen in blocks. Cryosections of 10 µm thickness were obtained using a Leica cryotome type CM3050S (Leica Biosystems, Wetzlar, Germany). Sections were stained with Safranin-O (VWR, Radnor, PE, USA) and Fast Green FCF (Sigma) to visualize glycosaminoglycans (GAGs) and collagen, respectively. Nuclear counterstaining was performed with Weigerts Hematoxylin A + B (Carl Roth, Karlsruhe, Germany). Additionally, frozen sections of the decalcified OE halves were stained with 1 µg/mL Propidium iodide (Sigma) to visualize nuclei and imaged using a Leica microscope. Images were acquired using a Leica DMi8 microscope (Leica, Wetzlar, Germany).

### 4.5. Biochemical Analysis of Cartilage Matrix Components

Cartilage portions isolated from OEs were carefully dissected using sterile scalpels, weighed and measured, and afterwards enzymatically digested in 140 µg/mL papain solution (Sigma-Aldrich) for 16 h at 60 °C, following established protocols by Kleuskens et al. [13]. DNA content was quantified using Hoechst 33258 dye (Lumiprobe, Hannover, Germany) with a standard curve generated from calf thymus DNA (Invitrogen, Waltham, MA, USA). Sulfated glycosaminoglycan (GAG) concentrations were determined via the dimethylmethylene blue (DMMB) assay, using chondroitin sulfate sodium (from shark cartilage, Sigma) as a reference standard [50]. Total collagen content was assessed by measuring hydroxyproline levels using a chloramine-T-based colorimetric assay, calibrated with trans-4-hydroxy-L-proline standards (Hydroxyproline Assay Kit, Sigma). Biochemical quantification of DNA and GAGs was normalized to the wet weight of the cartilage tissue and reported as micrograms per milligram (μg/mg) of wet tissue. In contrast, hydroxyproline content was normalized to the cartilage volume (μg/mm^3^) for analysis.

### 4.6. Statistical Analysis

All statistical analyses were conducted using GraphPad Prism version 10.2.3 (GraphPad Software, San Diego, CA, USA). Two-way analysis of variance (ANOVA) followed by a Dunnett’s post hoc test was employed to evaluate the effects of culture duration and defect treatment on DNA, GAG, and hydroxyproline content. Each OE was treated as an independent experimental unit (*n* = 3 per group), based on evidence that articular cartilage exhibits “significant site-specific variations” in ECM composition [51]. This biological heterogeneity justifies the assumption of independence between explants harvested from distinct locations within the same donor. For comparative statistical evaluation, a cultivated empty defect group served as the internal reference control, representing the intrinsic repair capacity of untreated osteochondral defects under standardized in vitro conditions. This group provided the baseline against which the efficacy of therapeutic interventions was assessed, both in absolute terms and as a percentage improvement over the untreated state (empty defect). Pearson correlation analysis was used to assess linear correlations between biochemical parameters across treatment groups. A *p*-value of *p* ≤ 0.05 was considered statistically significant.

## 5. Conclusions

This study underscores the translational relevance of a standardized human ex vivo OEs model as a feasible platform for elucidating the spatial and temporal dynamics of cartilage repair. The persistence of metabolically active MSCs and their spatial association with nascent matrix deposition supports their functional involvement in early tissue remodeling, particularly within permissive scaffold environments. The collagen type I hydrogel facilitated a coordinated cell–matrix response suggestive of chondrogenic engagement, whereas fibrin and MSC-filled OEs revealed a more heterogeneous regenerative trajectory, possibly reflecting matrix-induced variability in MSC behavior. Nevertheless, the consistently attenuated correlations relative to native tissue highlight the partial reconstitution of homeostatic architecture within the studied timeframe. These findings not only validate the OE’s capacity to differentiate between scaffold effects but also emphasize the importance of extended observation periods, increased experimental power, and cellular fate mapping to resolve the complex interplay between biomaterials, cell populations, and regenerative outcomes.

## Data Availability

The original contributions presented in this study are included in the article. Further inquiries can be directed to the corresponding author.

## Abbreviations

ECM—extracellular matrix, GAG—glycosaminoglycans, MSC—mesenchymal stem cells, MTT—3-4,5-dimethylthiazol-2-yl-2,5 diphenyl tetrazolium bromide, OEs—osteochondral explants.
